# Kinematics of the viscous filament during the droplet breakup in air

**DOI:** 10.1038/s41598-022-05839-y

**Published:** 2022-02-02

**Authors:** Diana Broboana, Ana-Maria Bratu, István Magos, Claudiu Patrascu, Corneliu Balan

**Affiliations:** REOROM Laboratory, Faculty of Energy Engineering, University Politehnica of Bucharest, Splaiul Independentei 313, 060042 Bucharest, Romania

**Keywords:** Engineering, Physics

## Abstract

The dripping regime in the vicinity of the fluid droplet breakup is analyzed using the correlation between experiments and numerics. The evolutions of filament’s neck and its corresponding thinning velocity are described using the logistic functions. Three flow regions are observed as the relative time decreases: (1) a monotonous increase of the neck’s thinning velocity, where inertia and capillarity are balanced, (2) a transition domain characterized by the equilibrium between inertia, capillarity, and viscous forces, where the thinning velocity varies non-monotonically with the relative time and (3) the final droplet pinch-off, where velocity decreases or oscillates around a constant value. The distributions of the $\upzeta$-coefficient (parameter related to the non-dimensional second invariant of the velocity gradient) on the filament’s surface and droplet’s profile characterize the kinematics at the interface. The regions dominated by extension, where pure elongation is located at $\upzeta \cong 1$, are determined. One main result of this study is the confirmation that distribution of the $\upzeta$-coefficient is a relevant parameter to analyze and to quantify the breakup process. This result has the potential of developing novel techniques and more precise procedures in determining the interfacial rheology of viscous and complex fluids.

## Introduction

The stretching of filaments and breakup of droplets have been intensively studied in the last decades. Past studies have shown that universal exponents emerge when using scaling functions that characterize the pinch-off process^1^. The pinch-off is directly related to droplet formation and its subsequent filament thinning stages^2–5^, most of them being correctly described by nonlinear theories^6^. If Weber number remains below 4, the injection of a fluid will manifest as a dripping regime. For low viscosity fluids, the transitions from dripping to jetting are characterized by the dependence between Weber and Bond numbers^7^. Other types of transitions have also been identified and associated with droplet formation or with the breakup process of a contracting liquid filament^8–12^.

One of the main target of the past studies was to establish the relation between the dynamics of the filament, from its formation to the droplet pinch-off, with the extensional properties of the fluid sample. The investigations of filaments thinning include: the rheology of transient filament thinning processes^13,14^, the dependence of the diameter profile on fluid properties^15,16^, the deformation of shear-thinning liquid bridges^17^, the capillary breakup of viscous and weakly elastic fluids using CaBER experiments^18^, the influence of an external liquid^19^, and the drop dynamics^20^. The applicative potential is related to material characterizations and the measure of relaxation time^16,21^, ink-jet printing technologies^22^, extensional rheology of suspensions and mixtures^23,24^, microfluidic devices^25,26^, and the influence of surfactants^27^. The break-up of liquid filaments and droplet pinch-off is still an actual field of study, even for Newtonian fluids. The experimental investigations performed with new setups correlated to numerical modelling of the flow dynamics are used today to confirm or not the theoretical predictions and to establish the limitations of the existing models of the threads thinning^12,28–30^.

The paper is concerned with the experimental investigations and the numerical modeling of droplet formation, and the breakup process of a viscous filament surrounded by air. The goal of the paper to determine the kinematics of a liquid filament, before the droplet detaches from a capillary needle. The study is focused on the evolution of the minimum diameter of the filament, the place where it breaks and the droplet pinch-off is produced. After the definition of a benchmark case of study (the formation and detachment of an oil droplet at constant velocity and Ohnesorge number close to unity) and the correlation of the numerical solutions with experiments, there are determined the filament’s profile and the local velocities distributions on the filament’s surface. The analyses of the data confirm the existence of the three regimes during the filament thinning. Except for the very vicinity of the pinch-off (where the regime is linear), the dynamics of minimum diameter is well approximated by a 4-parameters logistic function, for all the viscous tested samples (with exception of water). The proposed kinematics parameters to characterize the filament interface during thinning are: (a) the minimum diameter thinning velocity, and (b) the distribution of the $\upzeta$-coefficient on the interface and inside the liquid (a non-dimensional quantity related to the second invariant of the velocity gradient), which also quantifies the surface elongation and indicates the locations of possible regions with pure extensional flows. In the present study the experiments confirm the numerics and both corroborate the relevance of the defined kinematics parameters, which are directly linked to the flow regimes before the filament breakup.

## Methods

**Figure 1:**
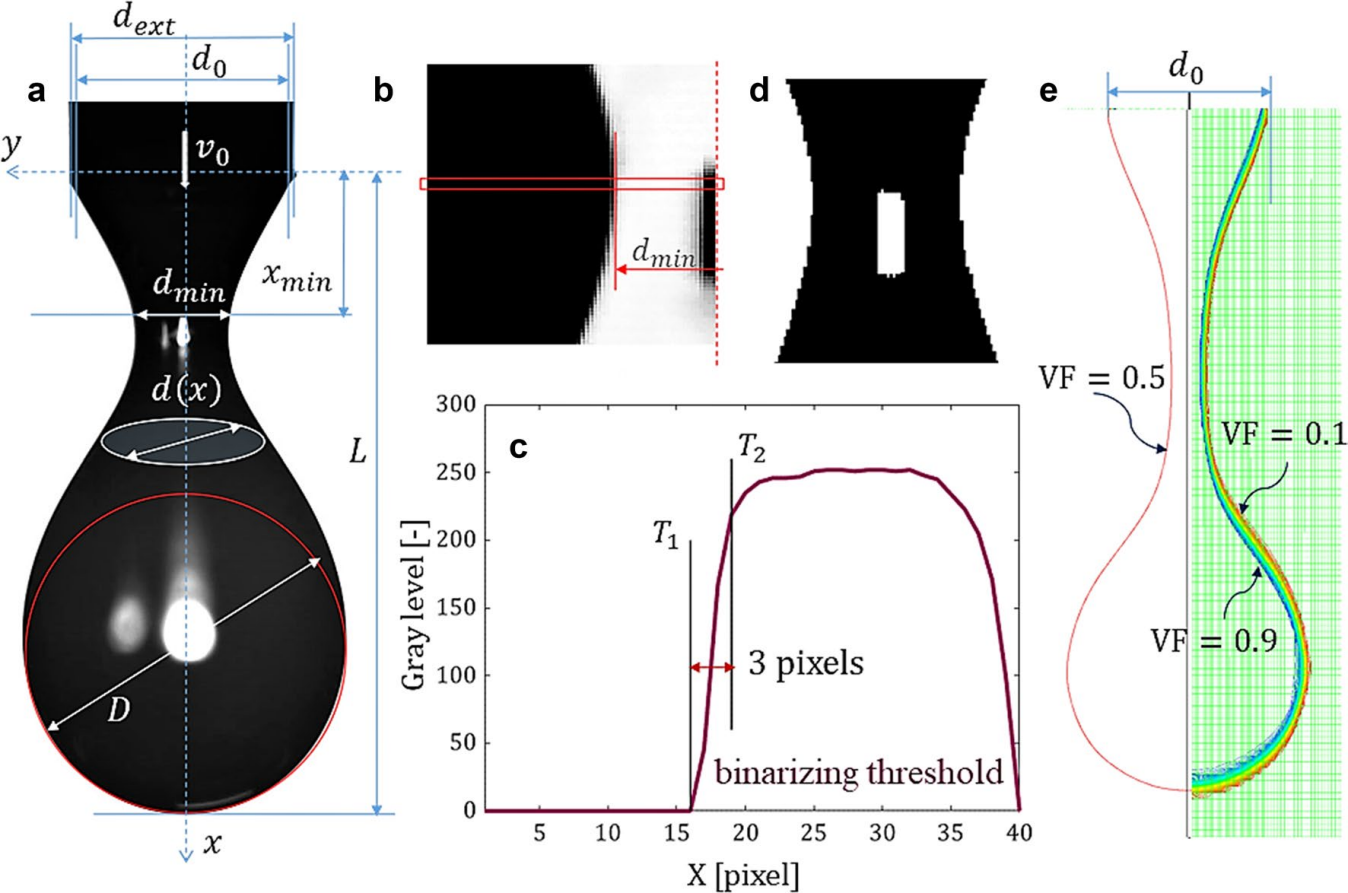
(**a**) Depiction of the investigated dimensions of a detaching droplet (silicone oil sample). Steps of the original imagine processing; detail of the neck diameter ${d\left(x\right)=d}_{min}$: (**b**) negative imagine, (**c**) distribution of gray levels on the inspection line, corresponding to the minimum thickness of the filament, (**d**) final binarized image. Numerical interface (**e**); the mesh/cell size is 27.5 μm and the VF $\in \left[0.1, 0.9\right]$ covers 3 cells. The median line VF = 0.5 approximates the computed interface with a maximum error of 42 μm.

The experimental and numerical investigations of the droplet formation process and the subsequent detachment from the capillary in air are performed in a confined geometry. The constant flow rate of the liquid sample is imposed in a capillary needle with the inner diameter ${d}_{0}=2.21$ mm using a Harvard syringe pump, the mean velocity ${v}_{0}$ inside the needle being maintained constant. The experiments were performed in the interval ${v_0} \in \left( {1 \div 20} \right)$ mm/s for different fluid samples: Newtonian (water, oil, glycerin), viscoelastic (saliva, PAA solution) and pseudoplastic (yield stress fluid). To avoid possible external perturbations due to the air current, the needle was fixed in the middle of a glass tank with 200 mm height and square cross section of 150 mm × 150 mm. As the liquid leaves the needle it forms a droplet which then detaches under its own weight, leaving behind a liquid filament that progressively thins until rupture. Snapshots of the phenomenon are then obtained using two high-speed cameras: Phantom VEO-E 340L (resolution of 1280 × 1000 pixels at 2000 fps, pixel size 10 microns) and FASTCAM mini-UX100 (1280 × 1024 pixels at 4000 fps, pixel size 10 microns). The scale of each picture is set by the outer diameter of the needle*,* ${d}_{ext}=2.7$ mm. The minimum neck diameter (${d}_{min}$), the distance between the apex of the drop and the needle ($L$) and the maximum droplet diameter ($D$), as shown in Fig. 1a, are then measured using the ImageJ or Matlab software, the two fluids in contact being identified with black and white colors, respectively. The pixel dimension corresponding to the ImageJ resolution is 20 µm. The errors in measurements appear due to the presence of the “grey” pixels at the boundary between the two fluids in contact (generated by optical interference/reflection). The image processing performed in the Matlab environment is based on a binarization technique (see Fig. 1a–d). To determine the binary threshold, a detail is extracted from the area of the filament interface from the original image (Fig. 1a). The obtained image is negativated (Fig. 1b) and the line of pixels corresponding to the minimum diameter of the filament is represented graphically (Fig. 1c) to observe the transition more easily from background to object. It is considered that the interface starts at the pixel with the first value other than 0 and ends at the pixel whose value is 50% less than that of the next pixel. The two determined values (T_1_ and T_2_, respectively) represent the ends of the range that includes the pixel values at the interface. The binarization threshold is considered the arithmetic mean of the ends of this interval. The final image (Fig. 1d) is obtained by binarization of the original image with the found threshold. The band between T_1_ and T_2_ covers 3 pixels, therefore the error in determining the interface is approximately 30μm.

Non-stationary solutions of the Navier–Stokes equations (Newtonian fluids under incompressible and isothermal conditions) for the whole flow domain are obtained with the commercial Fluent code, the dynamics of the droplet interface being computed using the VoF model^31–34^. The geometry is axial-symmetric and contains 378,800 quadrilateral cells and 380,460 nodes. The time step is 0.1 ms with 400 maximum iterations per time step at 10^–8^ precision. The laminar solver for the momentum equations is based on the Quick upstream interpolation scheme. The Courant number is maintained below 0.5 and the values for the cell Reynolds number are below one, therefore the spurious oscillation of the numerical solution are absent, and the calculus accuracy is satisfactory^35,36^.

The working PC has 16 parallel processors at 3 GHz and 128 GB RAM memory. For one case, the necessary computation time to obtain the rupture/detachment of the jet/droplet from the capillary is at least 25 days. The boundary conditions are (1) no slip at the solid wall, (2) free surface at the upper limit of the domain, (3) constant velocity at the entrance of the capillary, (4) a 90° contact angle between the tested fluid and the capillary wall (considered with zero thickness). The interface between the immiscible fluids was traced using the VoF implicit scheme at a volume fraction (VF) of 0.5. The errors in the measurements of the interface profile are given by the width band between VF = 0.1 and VF = 0.9, which for thin filaments covers almost 50% from the nominal measured dimension, Fig. 1e. However, the limit of our calculus is established by the mesh quality (the number of nodes/cells) and directly related with the computation time step and the available resources. The error of drawing the computed interface is almost 50 μm, a similar value with the numerical modeling of the capillary breakup of a liquid bridge^30^.

The benchmark case is defined by the flow of a viscous Newtonian silicon oil with velocity ${v}_{0}=10$ mm/s, and the following material properties (measured at temperature of 23 °C): density—$\rho =870$ kg/m^3^, viscosity—$\eta =0.2$ Pas and surface tension—$\sigma =0.025$ N/m. The flow dynamics is characterized by the following non-dimensional parameters: Bond—$Bo= \rho g{d}_{0}^{2}/\sigma \cong 1.65$, Reynolds—$Re=\rho {v}_{0}{d}_{0}/\eta \cong 0.1$, Weber—$We=\rho {v}_{0}^{2}{d}_{0}/\sigma \cong 0.0076$, capillary—$Ca=\eta {v}_{0}/\sigma \cong 0.08$, Ohnesorge—$Oh : = \sqrt {Ca/Re} \cong 0.9$, the process being scaled by the viscous and capillary times: ${t}_{v}=\eta {d}_{0}/\sigma \cong 0.017$ s and ${t}_{\sigma }=\sqrt{\rho {d}_{0}^{3}/\sigma }\cong 0.019$ s, respectively. Here ${d}_{0}$ and ${v}_{0}$ were considered the space scale and the velocity scale, respectively.

## Results and discussions

### The experimental and numerical droplet profiles

In the present work the analysis is focused on the benchmark case (droplets evolutions for different tested samples and flow conditions are displayed in Additional information AI.1–3). In this dynamical process, the capillary force dominates inertia ($$Ca, We$$ are much less than one) being balanced by the mass force ($$0.5 <Bo<1.5).$$ Viscosity is equilibrated by the capillarity ($$Oh$$ almost equal to unity) and inertia is not too relevant for the dynamics ($$Re<1$$). Therefore, we have a well-defined dripping regime, in the vicinity of the Stokes flow. Accordingly to the self-similar solution of the Stokes jet flow^2–4^, it is expected to find in the very vicinity of the rupture a linear increase of the minimum diameter of the filament ($${d}_{min}$$ in Fig. 1a) with the relative time: $$\tau : = {t_c} - t$$, where $${t}_{c}$$ is the time of the pinch-off (i.e. rupture time of the filament) and $$t$$ is the current time. However, the scaling dependence of $${d}_{min}$$ with $$\tau$$ depends on the $$Oh$$ number and the equilibrium between the viscous and capillary forces is perturbed by inertia (especially if the thickness of the filament is not thin)^7,12^. In our experiments the viscous time and the capillary time are almost equal, being considered appropriate scales for the process^12^; the time value in the range of 17–20 ms indicates the duration of the linear regime previously the pinch-off (dominated by viscosity and capillarity).

**Figure 2:**
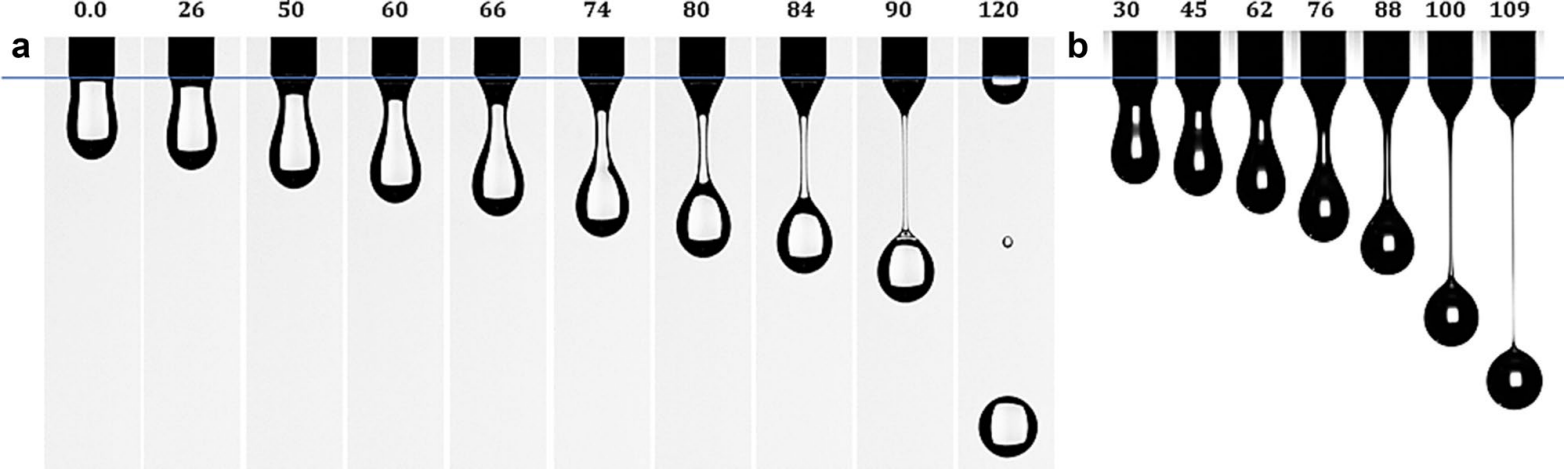
Dynamics of the droplet formation and the evolution of the neck filament of silicon oil in air at $${v}_{0}=10$$ mm/s; (**a**) experiment E1 (2000 fps), (**b**) experiment E2 (4000 fps). The reference droplet configuration ($$t=0)$$ corresponds to the first picture in the sequence (**a**), the images being captured from the movies at different time in the interval $$t\in \left[0, 120\right]$$ ms. The recorded *L*—length in time is almost identically for the experiments and numerics (see also AI.1).

**Figure 3:**
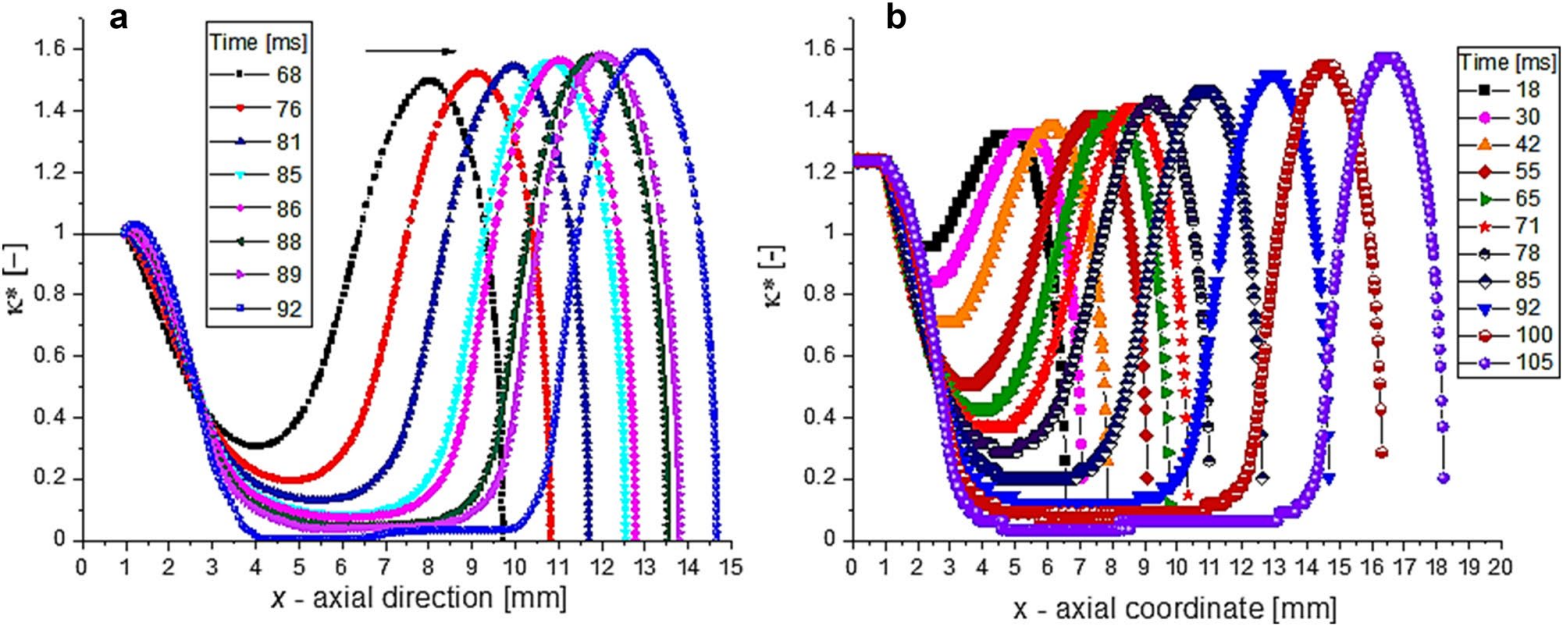
The contours of the droplet at different times, before the detachment from the needle: (**a**) numerical solutions at VF = 0.5, (**b**) experiments E2, Fig. 2b.

The dynamics of the droplet’s profile and filament thinning are displayed in Fig. 2a,b (series of experiments were performed at different velocities of the images acquisitions). The droplet pinch-off (the rupture of the filament, respectively) was observable in the time interval $t\in \left[100-110\right]$ ms from one established reference configuration ($t=0$ in Fig. 2a). The evolutions of the droplet contours are shown in Fig. 3a,b, where ${\kappa }^{*}\left(x\right)=d(x)/{d}_{0}$ is the non-dimensional local diameter, Fig. 1a. We have to remark that in numerical simulations the thickness of the needle was not considered, ${\kappa }^{*}\left(0\right)=1$ in Fig. 3a. In almost all experiments the fluid wets the needle; therefore, the initial fluid diameter of the experimental data is ${d}_{ext}=2.7$ mm, respectively ${\kappa }^{*}\left(0\right)=1.22$ in Fig. 3b. Consequently, in experiments the filament pinch-off is expected to be delayed in comparison with the numerical simulations.

### Time variation of the minimum diameter and thinning velocity

**Figure 4:**
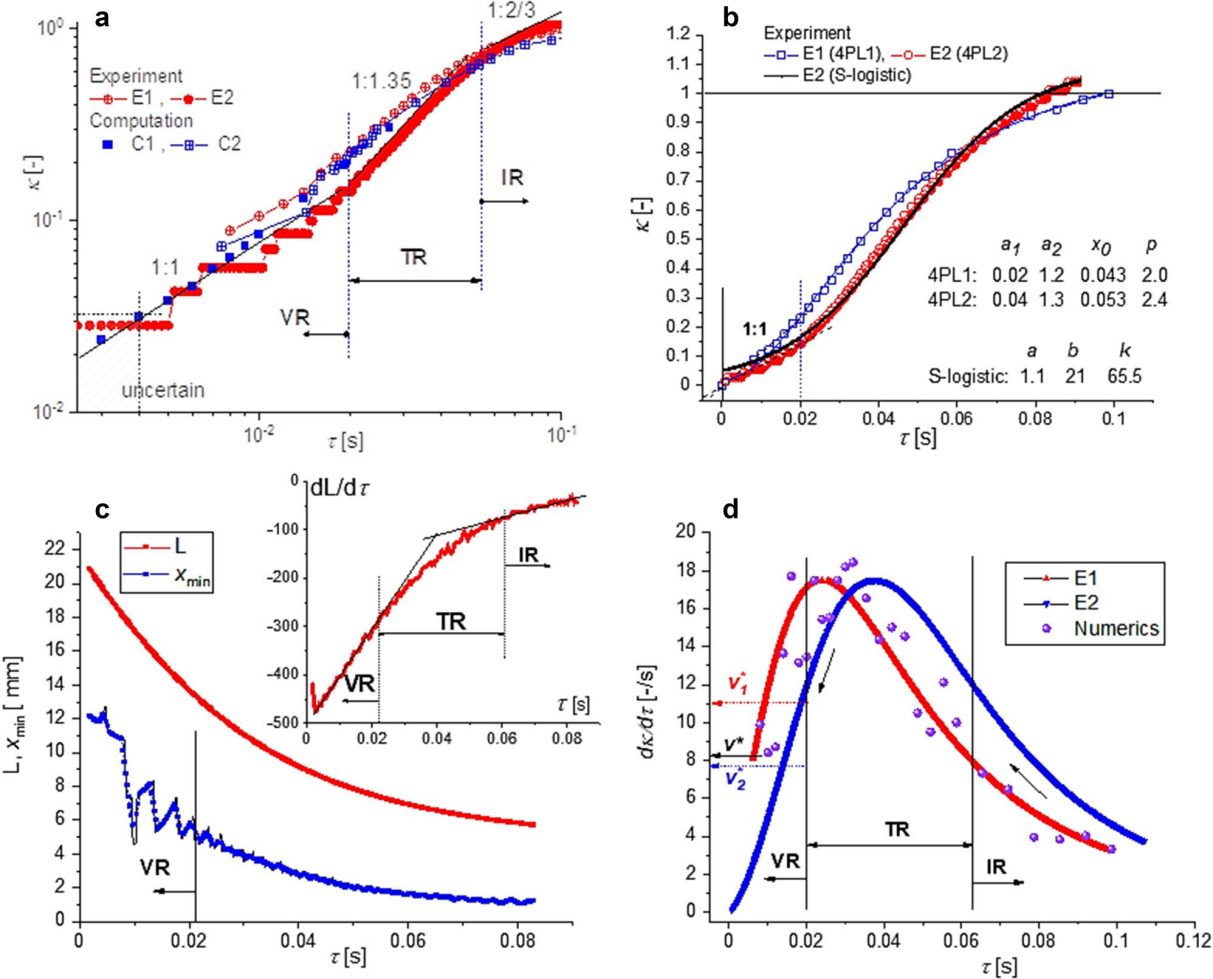
(**a**) Minimum neck diameter ${\kappa =d}_{min}/{d}_{0}$ as function of the relative time $\tau$ (experiment and numerics from Fig. 2), the scaling of $\kappa$ with $\tau$ in the 3 flow regimes: VR (linear)—viscous, TR—transitory, IR—inertial; (**b**) fitting of the experimental curves $\kappa \left(\tau \right)$, see AI.2 for comparison with data at ${v}_{0}=1$ mm/s; (**c**) variation of *L* and ${x}_{min}$ (see Fig. 1) for the experimental data E2; (**d**) the thinning velocity $d\kappa /d\tau$; the value ${v}^{*}\cong 8$ [–/s] is given by the formula^3,4^ $d\kappa /d\tau =2\cdot 0.0709/{t}_{v}$. Constant velocities ${v}_{1}^{*}$ and ${v}_{2}^{*}$ corresponds to the linear dependence $\kappa \left(\tau \right)$ from (**a**,**b**).

The evolutions of the minimum non-dimensional diameter ${\kappa \left(\tau \right)=d}_{min}/{d}_{0}$ are shown in Fig. 4a,b. The two sets of the experimental data are fitted in Fig. 4b with the S-curve represented by 4PL, the 4^th^—parameters logistic function $\kappa \left(\tau \right)={a}_{2}+\frac{{a}_{1}-{a}_{2}}{1+{\left(\tau /{\tau }_{0}\right)}^{p}}$ or with the S-logistic function $\kappa \left(\tau \right)=a\left(1+b\cdot {e}^{-k\tau }\right)$, where ${a}_{1}, {a}_{2}, {\tau }_{0},p$ and $a, b, k$ are constants (the values are given in Fig. 4b).

The choosing of 4PL function for fitting the data is suggested by the equilibrium of the normal stresses for a slender jet, where inertia is balanced by the normal extra-stresses difference and the surface tension^4^. Assuming that inertia force in proportional to the thinning velocity, the surface force is proportional with the area and the capillary force with the diameter, one results the equation: $d\kappa /d\tau ={c}_{1}{\kappa }^{2}+{c}_{2}\kappa$ with the classical S-logistic function as solution (${c}_{1}$ and ${c}_{2}$ are material/process coefficients assumed here to be constants). Of course, this relation is just a qualitative approximation of the local dynamics, but its solution offers a fair fitting of both the experiments and the 4PL function for $\tau >20$ ms. In that domain, up to the onset of the VR (linear)-regime, 4PL functions give a good representation on the experimental data for many fluid samples with viscosity greater than water, including viscoelastic solutions and yield stress fluids (see Additional information AI.3).

The relative time variations of *L*, ${x}_{min}$ and $dL/d\tau$ (corresponding to the experimental E2 data) are plotted in Fig. 4c. The experimental and numerical thinning velocity $d\kappa /d\tau$ are represented in Fig. 4d (the thinning velocity in relation to the symmetric liquid bridge breakup was also investigated in the numerical study by Li and Sprittles^30^, where it is called “speed of breakup”).

The variations from Fig. 4 disclose the existence of three flow regimes^12^: (1) inertial—capillary (IR) where the filament neck follows at the beginning the scaling $\kappa \sim {\tau }^{2/3}$, and the thinning velocity is increasing, (2) transitory (TR)—balance between inertia-capillary and viscous friction, where the thinning velocity reaches the maximum, (3) viscous—capillary (VR) linear regime characterized by the decreasing of thinning velocity to a constant value ($\kappa \sim \tau$) in the vicinity of the pinch-off. Similar dynamics were observed in different tested configurations, droplet detachment experiments^9–12^ or in CaBER rheometer^14–18^. It is remarkable the qualitative correlation between the dynamics of the length *L* and the minimum diameter $\kappa$, in both time dependences $\kappa \left(\tau \right)$, Fig. 4a, and $dL/d\tau \left(\tau \right)$, Fig. 4c, the flow regimes being well defined. We notice that thinning velocity reaches a maximum before the onset of the VR-regime, where the oscillation of ${x}_{min}$ starts to be amplified. It is generally admitted that in the final stage the thinning velocity is constant^2–4,7,12^. The validity of experimental and numerical results are limited by the space error in the detection of the interface’s profile and filament thickness (the error in our work is in the range of 30–40 μm, Fig. 1). Therefore, the present results lack of precision in the very vicinity of the pinch-off at $\kappa <0.03$ and $\tau <4$ ms. Hence, we cannot confirm or infirm in this case that previously to filament rupture the thinning velocity is constant.

### Kinematics of the filament’s elongation

Droplet formation at low Reynolds numbers, followed by droplet detachment and pinch-off generates (in confined symmetric geometries with symmetric boundary conditions) symmetric vortical structures inside the injected liquid and in the outer fluid^37^. These unsteady vortical structures are connected, deforming with the interface of the droplet, and diffusing in the whole surrounding fluid domain. One measure of vortex existence in a flow field is the magnitude of the local vorticity number $\mathcal{W}\mathcal{o}=\left|{\varvec{\Omega}}\right|/\left|\mathbf{D}\right|$, where ${\varvec{\Omega}}$ is the spin tensor and **D** is the strain rate tensor (i.e. vorticity number is the ratio between the local vorticity magnitude $\omega$ and the local strain rate $\dot{\gamma }$)^38^.

**Figure 5:**
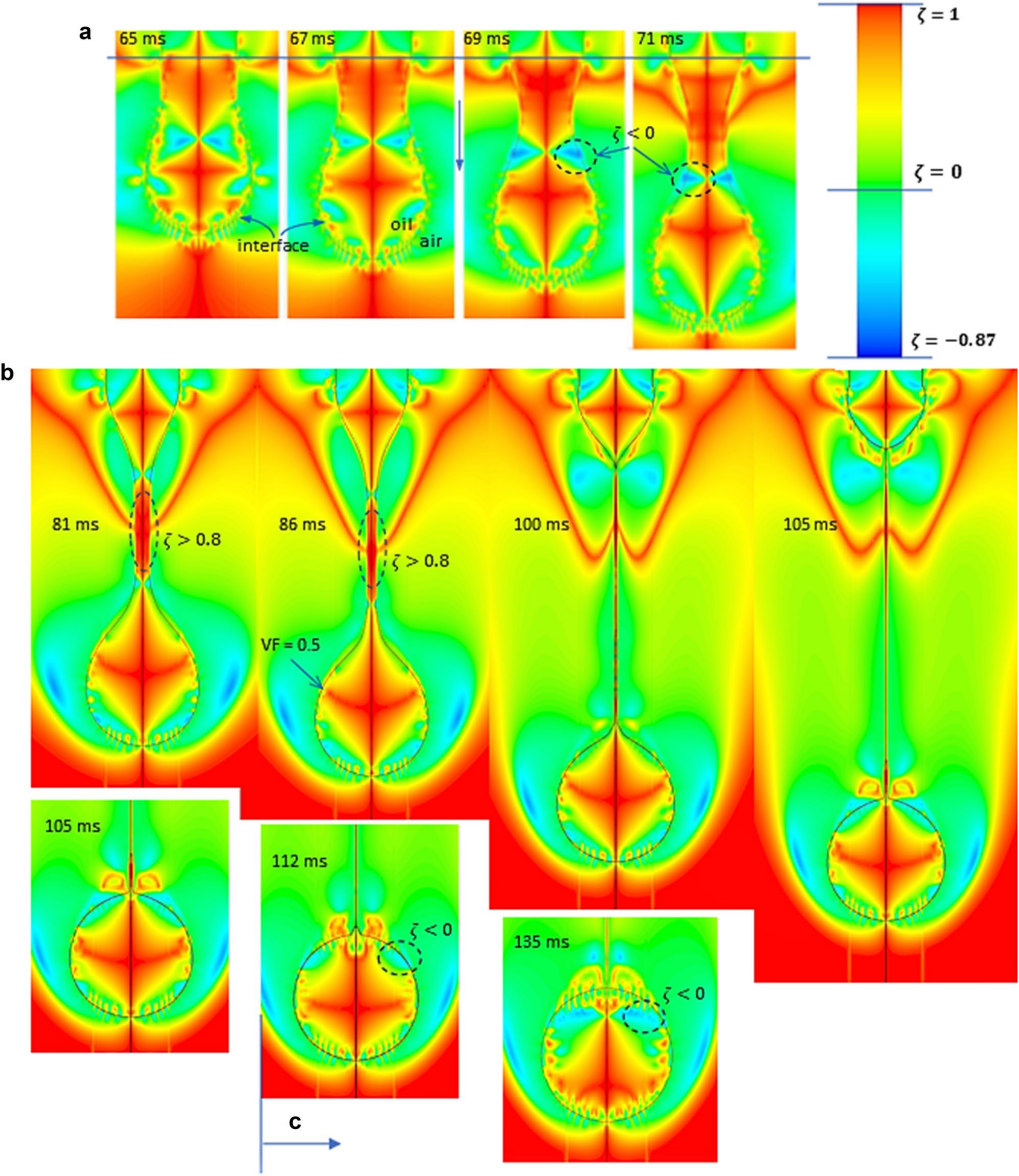
Distribution of $\zeta$-coefficient in the droplet and in its vicinity: TR-regime (**a**), VR (linear)-regime (**b**), post pinch-off (**c**). It is important to remark that vorticity dominant regions ($\zeta$ < 0) inside the droplet are almost absent in the VR regime (**b**), where extension is dominant (especially in the filament).

The necessary condition (but not a sufficient one!) for the existence of the vortex in a flow domain is $\mathcal{W}\mathcal{o} >1.$ The value $\mathcal{W}\mathcal{o}=1$ is valid not only at the solid walls or interfaces in contact with pure viscous fluids (where the adherence condition is valid), but also in simple shear motions (included in the class of flows with solenoidal acceleration). There are some other criteria to characterize and to quantify the local flow kinematics, one being the $\zeta$-coefficient^39^,$\zeta =(1-\mathcal{W}\mathcal{o})/(1+\mathcal{W}\mathcal{o}$). The $\zeta$-coefficient takes any value in the interval − 1 to + 1: (1) $\zeta = -1$ defines a pure rotation, (2) $\zeta =0$ indicates a simple shear motion and, (3) $\zeta =+1$ defines a pure extensional flow. In the present study $\zeta$-coefficient is used as a criterion to detect the flow region on the interface where extension is dominant (Fig. 5)^40^.

**Figure 6:**
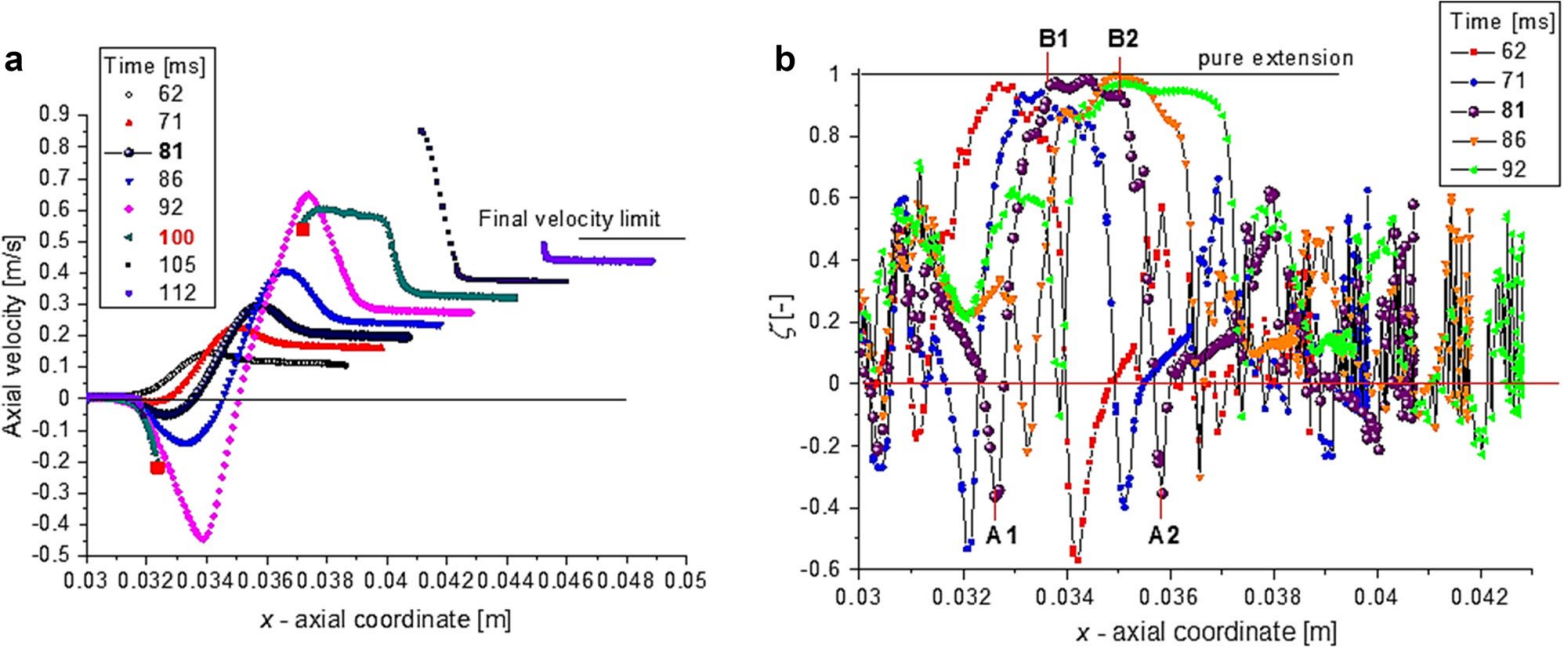
(**a**) Axial velocity ${v}_{ax}={v}_{x}$ and (**b**) $\zeta$-coefficient distributions on the droplet/filament interface at different values of time (at *t* = 81 ms the curves are bolded).

**Figure 7:**
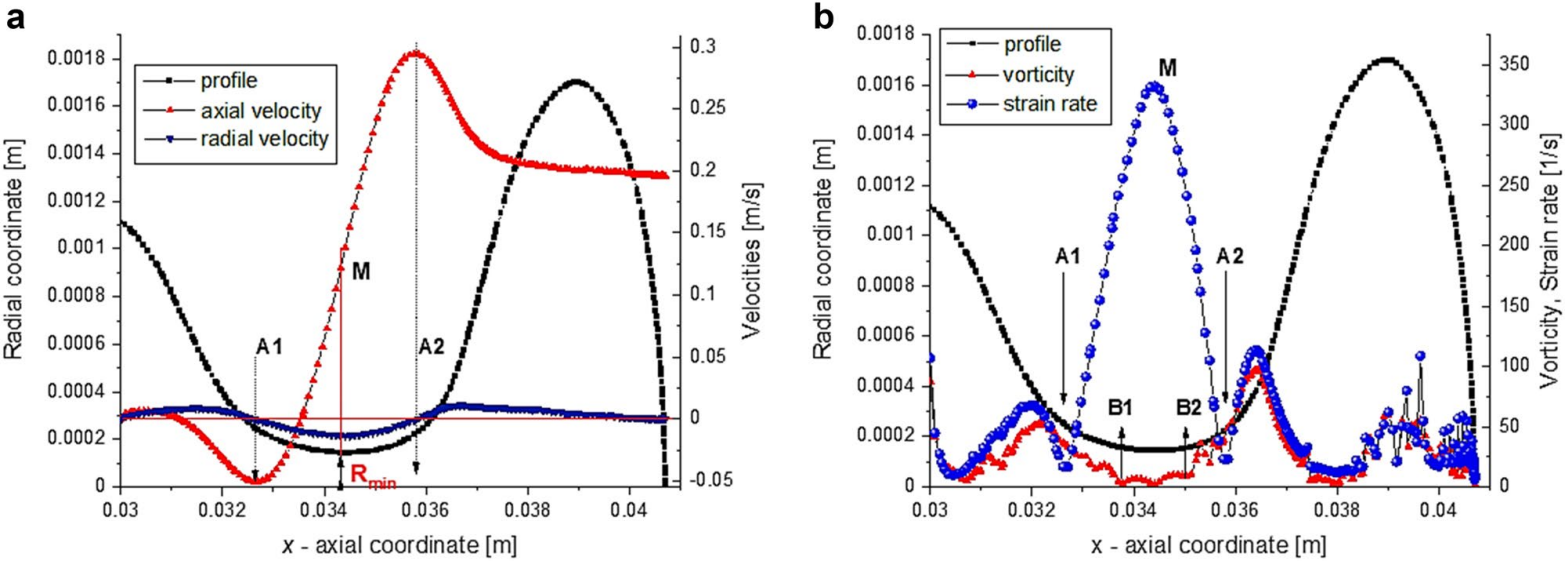
(**a**) Velocity components, (**b**) the vorticity and strain rate, along the droplet interface at time *t* = 81 *ms*.

The axial velocity distribution ${v}_{ax}\left(x\right)$ on the interface from Fig. 6a indicates the rupture time, i.e. $\tau =0$, in the vicinity of $t=100$ ms(see Fig. 2). As we observed from Fig. 4, the transition between TR and VR regimes takes place around $t\cong 80$ ms ($\tau \cong 20$ ms). The pattern of $\zeta$- coefficient from Fig. 5 is different in the two regimes. In TR-regime, $t<80$ ms (Fig. 5a), the vortical regions (defined by $\zeta <0$, blue color) are present in the liquid phase (inside the droplet). Once the dynamics enters in the last stage, $t>80$ ms, the vortical regions can be observed in air and in the very vicinity of the filament’s limits, Fig. 5b. The $\zeta$-distributions from Fig. 6b disclose that pure elongation ($\zeta \cong 1$) is reached almost in the middle of the filament (expected result, but now confirmed by numerics), the extension dominant region ($0<\zeta <1$) being limited by local vorticity concentration ($\zeta <0$). A detailed analysis of the local kinematics of the interface at time $81 ms$ (at the onset of the VR-regime) is shown in Figs. 6b and 7a,b. The filament is limited by the extremes values of the axial velocity ${v}_{ax}$, where radial velocity ${v}_{rad}$ and strain rate are zero (A1 and A2 in Fig. 7a,b). The minimum filament thickness corresponds to the maximum strain rate and to the inflection point in axial velocity distribution, respectively (point M in Fig. 7a,b); it is in the region where vorticity is minimum (Fig. 7b) and $\xi$-coefficient is close to unity (between the points B1 and B2 in Fig. 6b). The vortical structures are present at the interface in the vicinity of points A1 and A2, where vorticity is maximum (Fig. 7b) and $\xi$-coefficient reaches minimum (negative values) (Fig. 6b).

The numerical simulations are qualitatively and quantitatively confirmed by the experiments, the results being coherent and consistent with the previous studies dedicated to the filament break-up phenomenon. The analyzed case discloses that thinning velocity of the filament reaches maximum at the onset of the viscous regime and the evolution of the minimum diameter of the filament against the relative time is well fitted by a S-curve (not confirmed in the very vicinity of the filament rupture). Using the computed kinematics, the regions where the motion is close to pure extension on the surfaces of the filaments are precisely located. From the numerical results one can be computed two Reynolds numbers which define the thinning process: (1) ${Re}_{m}=\rho {v}_{m}{d}_{min}/\eta$*,* where ${v}_{m}$ is the axial velocity corresponds to the minimum diameter (at ${x=x}_{min}$) and (2) the local Reynolds number*—*${Re}_{local}=\rho v{^{\prime}}x{^{\prime}}/\eta$*,* where ${x}^{{\prime}}={{x}_{1.2{\cdot x}_{min}}-x}_{min}$ and ${v}^{{\prime}}$=${v}_{ax}\left(x{^{\prime}}\right)$, parameter introduced by Castrejón-Pita et al.^12^. The two Reynolds numbers have opposite monotonous variations during the filament’s thinning previous to the breakup. In the transitory and viscous regimes ${Re}_{m}$ is decreasing and ${Re}_{local}$ is increasing, with the following values in the vicinity of pinch-off (at $\tau =0.006 s$ and $\kappa \cong 0.04$, Fig. 4a): ${Re}_{m}$ = 0.0095 and ${Re}_{local}$= 6.52, respectively. It is important to mention that onset of viscous regime is well defined by a sharp change of the time derivative for both Reynolds numbers. In the viscous regime, at $\tau <0.02 s$, ${Re}_{m}<0.1$ and ${Re}_{local}>1.$ The values of ${Re}_{m}$ indicates the presence of the Stokes flow before rupture, which support the existence of a constant thinning velocity in that regime.

## Concluding remarks

The present work investigated the formation and breakup of viscous droplet in air. The main goal of the study is to characterize the flow kinematics on the surface of the filament, with the aim to establish the region with maximum extension. The main parameter followed in our investigations was the evolution of the filament neck in the vicinity of the droplet pinch-off. The numerics offer a good representation of the phenomenon. The computations are consistent with experiments, except for the region in the very vicinity of thread rupture, where the errors in the measurements and computations have the magnitude of the filament thickness.

Some relevant remarks follow the analysis of the thinning of the minimum filament diameter (i.e. the dependence of the non-dimensional diameter $\kappa$ against the relative time $\tau$): (1) a monotonous increasing of the neck thinning velocity where inertia and capillarity are balanced, followed by (2) a transition regime characterized by the equilibrium between inertia, capillarity and viscous force, where thinning velocity reaches the maximum value and varies non-monotonic, and (3) the final pinch-off regimes, where velocity is decreasing or oscillates around a constant value. The decreasing of $\kappa \left(\tau \right)$ function is well approximated by logistic functions, the S-curves fit almost perfect the data up to the vicinity of the filament’s rupture, where the regime is linear (however, in this region the uncertainty of the filament profile is high). Except for very low Newtonian viscous fluids (water), all the performed tests disclose the same evolution of $\kappa \left(\tau \right)$ function (experiments performed with different liquid samples are presented as Additional information in AI.3).

Once the numerical calculations are validated by the experiments, relevant information can be obtained about the kinematics of the investigated flows on the interface: (a) the distributions of velocities and strain rates, (b) the location of the vortical structures and vorticity magnitude, (c) the regions where pure extension is present. In this paper we emphasize the importance of the $\zeta$-coefficient distribution on the droplet/filament profile. The process of breaking is dominated by elongational deformation; in the vicinity of the pinch-off $\zeta$ > 0 on the filament surface and inside the droplet. The analysis of $\zeta$-coefficient distribution on the interface reveals the regions where the process is almost pure extensional ($\zeta \cong 1$), which coincide with a domain around the minimum thickness of the filament.

Finally, one concludes that $\zeta$-coefficient can be considered a relevant parameter to analyze the kinematics of interfaces; in particular, to detect the location on the separation surface between immiscible fluids where extension is dominant. The results have potential in developing novel techniques and more precise procedures in measuring the interfacial rheological properties of viscous and complex fluids in elongational flows. The time variation of the thinning velocity and the position of its maximum value relative to the moment of the filament rupture, correlated with the space distribution of the $\zeta$-coefficient (if the numerics is available), might be related to the material surface properties of the fluid (as surface/interfacial tension and surface viscosity^41^) and to the corresponding bulk Ohnesorge and local Reynolds/Elasticity numbers, respectively.

## Supplementary Information

Supplementary Information.
