# Supplementary material for: Kinematics of the viscous filament during the droplet breakup in air
Source: Sci Rep. 2022 Feb 2;12:1774. doi: 10.1038/s41598-022-05839-y (PMC8810882; doi:10.1038/s41598-022-05839-y)
Supplement: Supplementary file 1 — Supplementary Information. [file 41598_2022_5839_MOESM1_ESM.pdf]

Additional Information

Paper: Kinematics of the viscous filament during the droplet breakup in air, by Broboana D. et al.

AI.1 Correlation between experiments E1 and numerical simulation (complementary to Fig. 2)

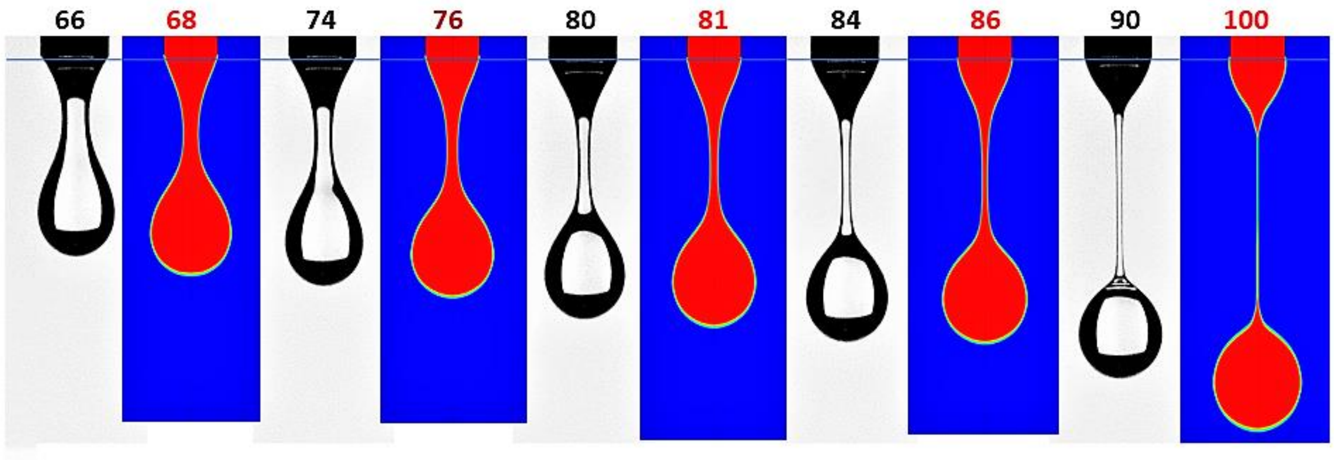

Figure AI.1 Comparison between sequences of experiment E1 (Fig. 2.a) and the numerical simulations (time scale [ms]).

AI.2 Experimental curves as function of the velocity v_0 (complementary to Fig. 4.b)

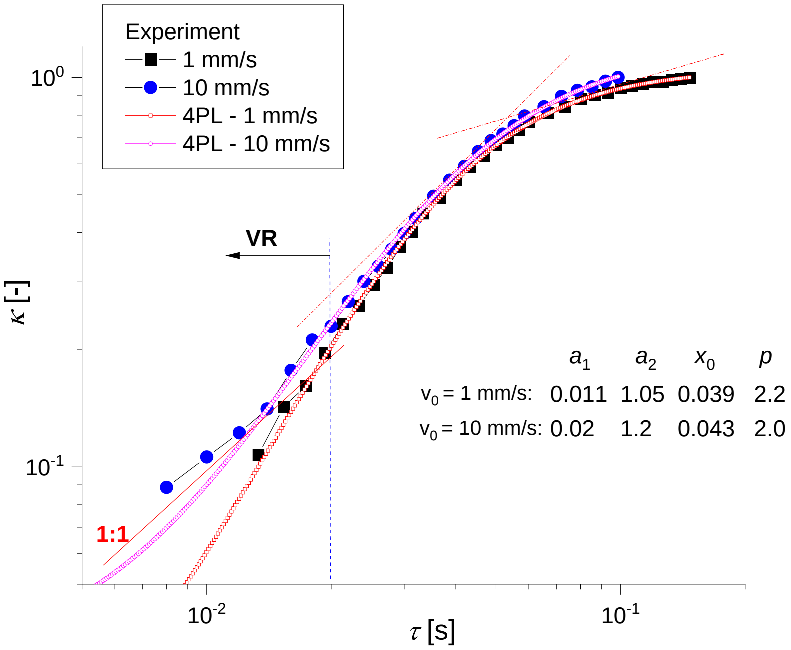

Figure AI.2 Fitting of the experimental curves $\kappa(\tau)$ for silicon oil visualizations (2000 fps) at two input velocities.

AI.3 Experiments with different fluids (complementary to Fig. 2 and Fig. 4.b)

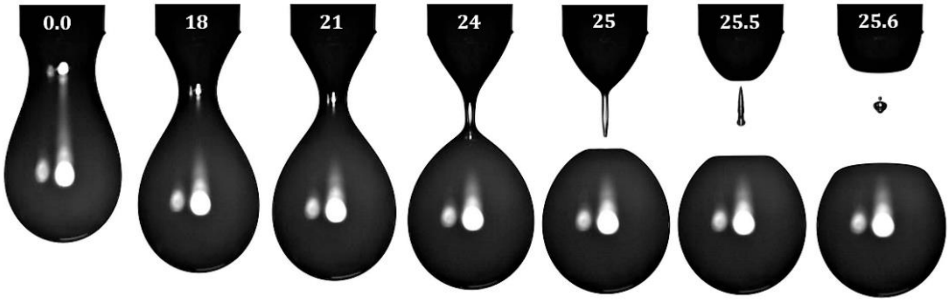

Figure AI.3.1 Dripping of water droplet in air in air
(visualizations at 4000 fps and $v_0 = 10$ mmm/s, $Oh_w \cong 0.0025$, time scale [ms]).

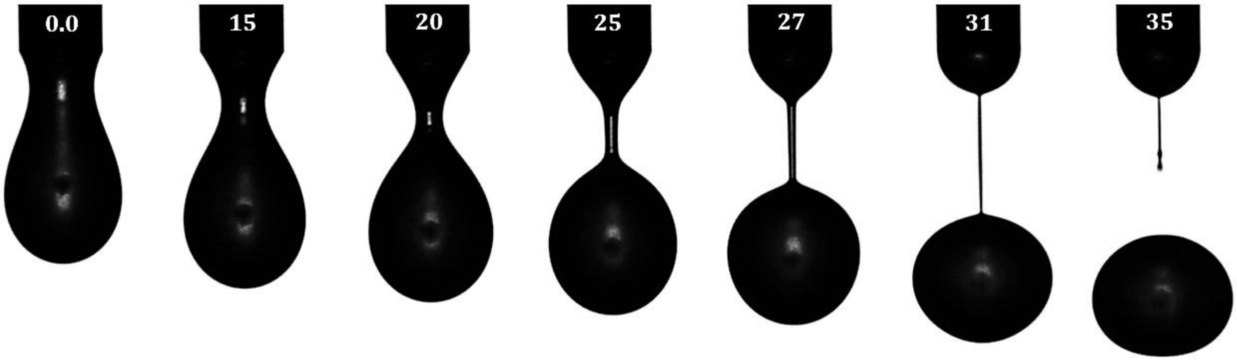

Figure AI.3.2 Dripping of saliva (viscoelastic fluid) droplet in air
(visualizations at 4000 fps and $v_0 = 10$ mm/s, $Oh_s \cong 0.08$, time scale [ms]).

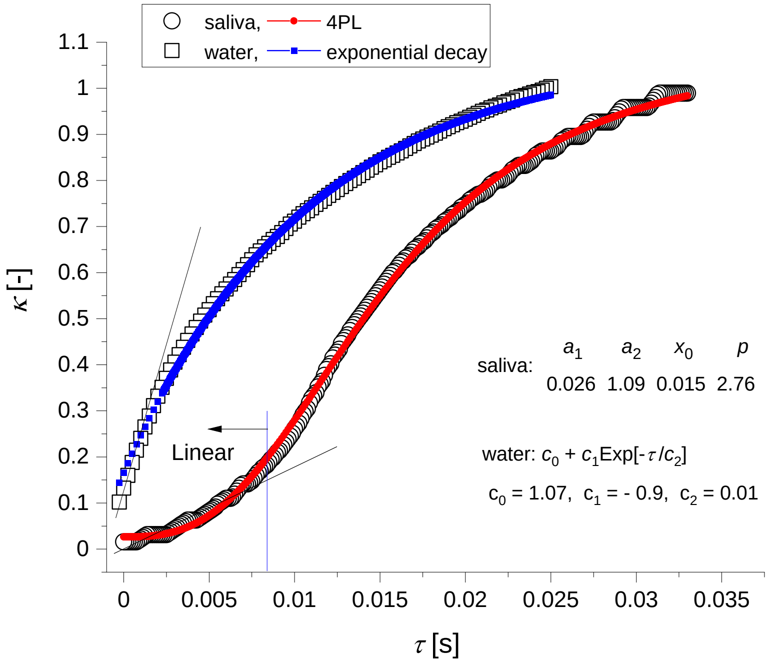

Figure AI.3.3 Fitting of the experimental curves $\kappa(\tau)$ for water and saliva samples.

Figure AI.3.4 Dripping of glycerin droplet in air
(visualizations at 8000 fps and $v_0 = 25\ mm/s, Oh_g = 2.47$, time scale $[ms]$).

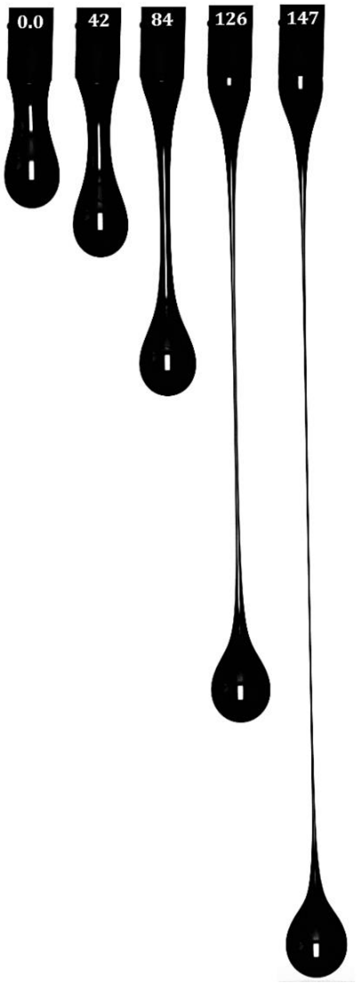

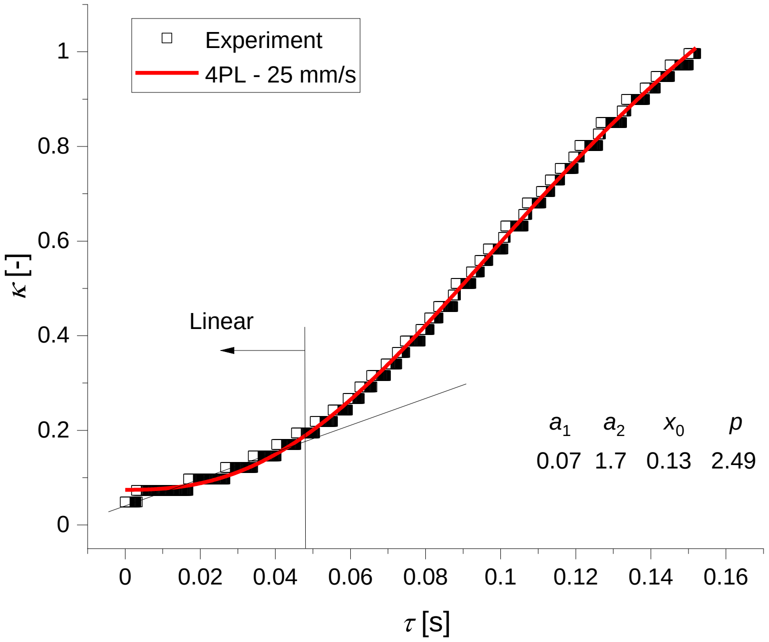

Figure AI.3.5 Fitting of the experimental curves $\kappa(\tau)$ of glycerin sample.

Figure AI.3.6 Dripping of PAA solution (viscoelastic fluid)
droplet in air (visualizations at 8000 fps and $v_0 = 10$ mm/s, $Oh_{PAA} \cong 0.18$, time scale $[ms]$).

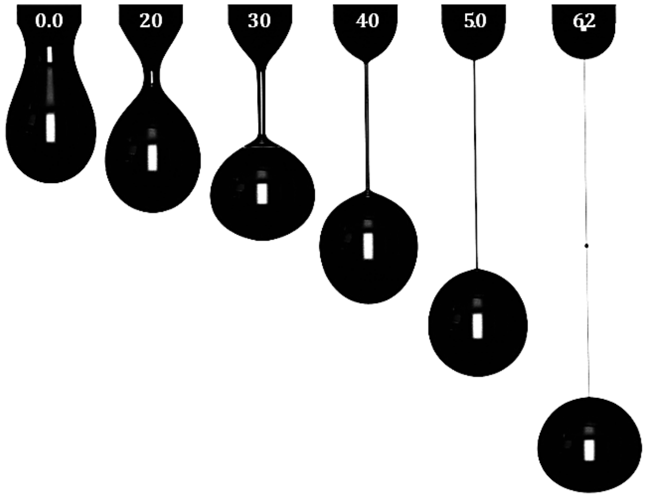

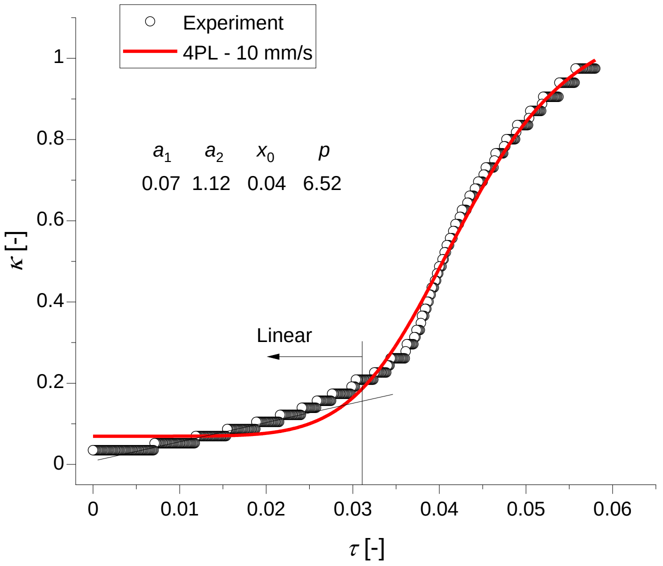

Figure AI.3.7 Fitting of the experimental curves $\kappa(\tau)$ for PAA solution.

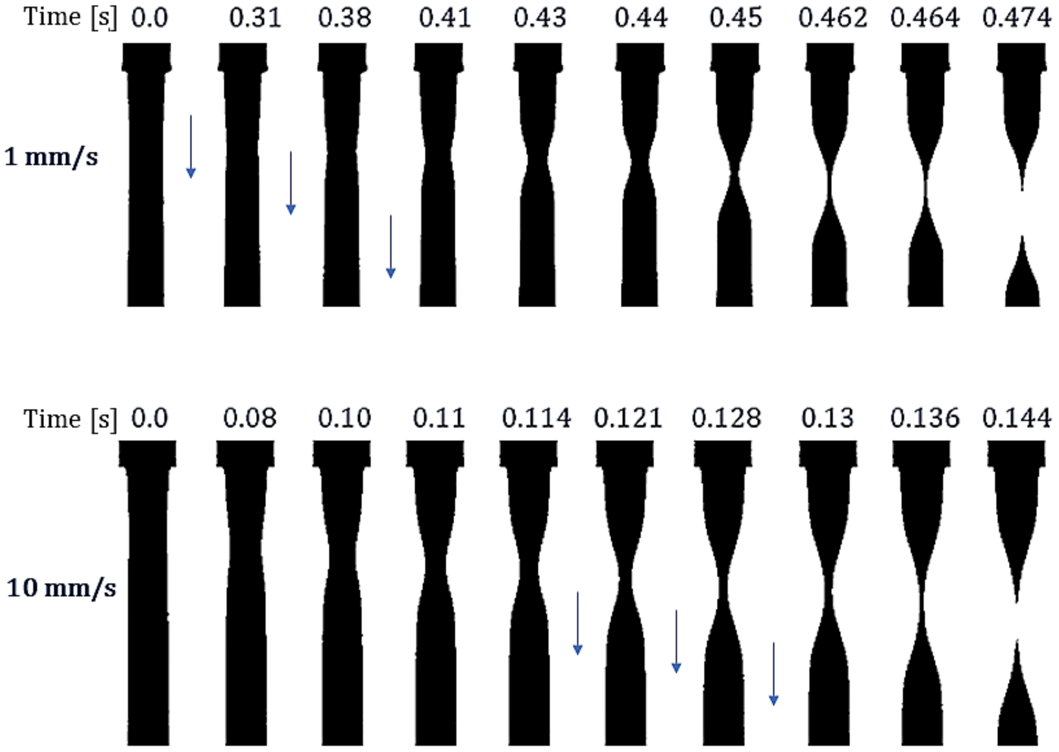

Figure AI.3.8 Thinning of cream's filaments (yield stress fluid; visualizations at 2000 fps, $Oh_c \cong 10^4$).

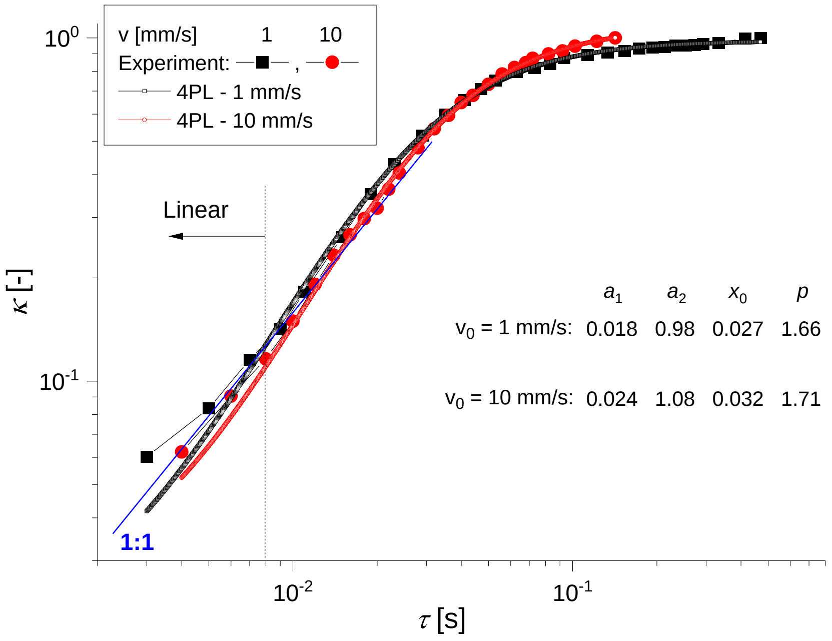

Figure AI.3.9 Fitting of the experimental curves $\kappa(\tau)$ for cream sample.
